# Nanotechnology-Based Drug Delivery Systems, 2nd Edition

**DOI:** 10.3390/pharmaceutics17010110

**Published:** 2025-01-15

**Authors:** Andrey N. Kuskov, Ekaterina V. Kukovyakina

**Affiliations:** Department of Technology of Chemical-Pharmaceutical and Cosmetic Substances, D. Mendeleev University of Chemical Technology of Russia, 125047 Moscow, Russia; kukoviakina.e.v@muctr.ru

## 1. Introduction

Nanotechnology is a promising and rapidly developing area in the 21st century, which affects various fields of science: physics, chemistry, biology, engineering, microelectronics, and medicine [1]. Nanotechnology is based on the use of materials that, when reduced in size to the nano-scale, acquire unique structural, optical, mechanical, magnetic, electrical, and biological properties [2,3]. In the field of medical nanotechnology, various nano-scaled structures have been developed on the basis of such materials, which can be used as carriers of drugs to improve their bioavailability, reduce systemic side effects, and increase therapeutic efficacy [4]. Such carriers must have a number of properties, such as bioinertness, biocompatibility, biodegradability, and a lack of toxicity to the organism, and must also protect the drug from degradation and inactivation [5] during transportation to the specific site, allowing for its prolonged release in the target area. In addition, effective medicine nanocarriers must be easy to obtain, reproducible, and affordable.

According to their origin, drug delivery systems can be divided into polymeric systems, cellular carriers and viruses, and inorganic carriers [6]. Polymer carriers include micelles, liposomes, dendrimers aggregates, hydrogels, etc. [7]. Cells in the body, such as immune cells (macrophages and T-cells), blood cells (erythrocytes and platelets), stem cells, exosomes, and adipocytes, can act as cellular drug carriers [8]. Finally, magnetic and metal nanoparticles, such as iron or gold oxide nanoparticles; carbon-based structures such as fullerenes, graphene sheets, and carbon nanotubes; quantum dots; and silicon dioxide-based nanoparticles are representatives of inorganic nano-scaled carriers for different therapeutic and diagnostic agents [9].

Biologically active substances can be conjugated with a carrier or loaded in it [10]. Depending on the physicochemical properties of the drug molecules, different types of carriers are used for immobilization. For example, a hydrophobic agent, the proteasome inhibitor bortezomib, was encapsulated in polymeric nano-sized particles based on amphiphilic derivatives of poly-N-vinylpyrrolidone, consisting of a hydrophobic core into which the biologically active substance was loaded and a hydrophilic shell that promotes compatibility with the aqueous environment of the entire system [11]. In hydrophilic molecules, for example, the antibiotic tetracycline was loaded into a hydrogel structure based on carboxymethylcellulose and a block copolymer of N-isopropylacrylamide and acrylamide [12], and the antitumor drug doxorubicin was loaded into liposomes [13]. Thus, nanotechnology-based delivery systems can be designed for both hydrophilic and hydrophobic molecules, as well as for low-molecular- and high-molecular-weight biologically active substances.

Therapeutic preparations based on deoxyribonucleic acid (DNA) and ribonucleic acid (RNA) molecules (antisense oligonucleotides, small interfering RNA, etc.) also have delivery features that can be enhanced using nanotechnological drug forms. Since DNA and RNA are negatively charged, for their successful loading into the delivery system, the carrier structure must contain positively charged groups to ensure an electrostatic interaction between DNA/RNA and the carrier occurs. The most common carriers for such purposes are liposomes and lipid nanoparticles consisting of ionizable or cationic lipids as the main component (in addition to cholesterol, phospholipids, and polyethylene glycol (PEG) lipids), as well as polymer carriers: poly(amidoamine) dendrimers and nanoparticles containing positively charged amino groups in their structure [14,15]. Thus, in Reference [16], plasmid DNA encoding glycoproteins of Rift Valley fever virus was successfully loaded into nanoparticles based on amphiphilic derivatives of poly-N-vinylpyrrolidone modified with the amino acids β-alanine or glycine.

Another perspective and a promising way to increase the therapeutic effect is the immobilization of several different drugs into the nano-scaled delivery system at once, which simultaneously affect different targets and have a synergistic effect. For example, cytarabine and daunorubicin (Vyxeos), when loaded into liposomes, showed increased efficacy in the treatment of acute myeloid leukemia compared to individual drugs [17].

Another key feature of nanotechnology-based carriers is that they can deliver biologically active molecules to the tumor area due to their enhanced permeability and retention (EPR) effect. However, this effect only contributes to the accumulation of carriers in tumors, and insufficient intracellular absorption remains a serious problem for effective chemotherapy. To improve and adjust the EPR effect, the surface of the nanocarriers can be modified with a target ligand, which is responsible for the directional transport of the drug in organism. Peptides, antibodies and their fragments, proteins, small molecules, and aptamers can act as ligands, which can selectively bind to receptors or antigens overexpressed on the cell surface, promoting the enhanced accumulation of nanocarriers in tumor tissue and accelerated integration by tumor cells [18,19]. For example, the iRGD peptide (CRGDKGPDC) is known for its ability to penetrate and accumulate in deep layers of the tumor, and its conjugation with nano-scaled carriers is a promising approach in cancer therapy [20].

Another approach in nanotechnological drug delivery is physically impacting the carrier structure. Physical factors include changes in pH, oxidation-reduction potential, temperature, ultrasound, magnetic field, etc. The essence of this method is that the carrier material reacts to a certain level of impact, leading to a violation of its integrity and, consequently, the intense release of the drug in the desired area [19,21].

However, to achieve the desired therapeutic effect, a sufficient number of drug-loaded nanoparticles must reach the target site. When entering the bloodstream, a key “obstacle” on the way to the target is the formation of the so-called protein corona on the carrier surface, which triggers the recognition of the nanoparticles by cells of the mononuclear phagocytic system (MPS) and their removal from the bloodstream. Therefore, to reduce the formation of the protein corona, increase the circulation time in the bloodstream, and avoid MPS capture, the surface of nano-carriers can be coated with neutrally charged polymers, such as polyethylene glycol, or other non-fouling materials, such as a cell membrane [22]. Another common option is MPS blockade, based on the saturation of macrophages with a blocking agent and the subsequent removal of this agent. This manipulation facilitates the introduction of the desired carrier, which will no longer be recognized by macrophages and therefore has the ability to circulate in the bloodstream for longer and accumulate in target tissues [23].

Some results obtained in the field of nanotechnology-based drug delivery systems have already been introduced into clinical practice; for example, liposomal forms of doxorubicin (Doxil and Myocet), daunorubicin (Daunoxome) [24], albumin nanoparticles with paclitaxel (Abraxane) [25], polymeric nanocarriers of doxorubicin (Transdrug), paclitaxel (Paclical), and neocarzinostatin (Zinostatin) [26].

However, each existing nano-delivery system has a number of limitations, such as its size range, the spectrum of drugs available for loading, the possibility of modification, its immunogenicity or scalability, the cost of production, and the rate of elimination by the mononuclear phagocytic system. This indicates the need to search for new alternative nano-scaled delivery systems for biologically active substances that are highly effective and safe for humans and animals, as well as technologically affordable, for their application as carriers of medicinal therapeutic and diagnostic agents.

## 2. Overview of the Published Articles

The five articles included in the current Special Issue, “Nanotechnology-Based Drug Delivery Systems, 2nd Edition”, draw attention to the importance of the development, investigation and application of nano-scaled drug delivery systems for novel medicines. The introduction of biologically active substances into various nano-delivery systems helps improve their physicochemical properties and stability, broaden their spectrum of action, provide a prolonged release, reduce toxicity, and enhance their therapeutic or diagnostic effect in vitro and in vivo.

Several articles in this Special Issue are devoted to the development and study of nano-scaled systems with immobilized biologically active substances for cancer (Contribution 1) and osteoporosis (Contribution 2) therapy. Luss A.L. et al. obtained nano-sized particles based on amphiphilic derivatives of poly-N-vinylpyrrolidone with encapsulated curcumin (Contribution 1). Loading curcumin into the hydrophobic core of the particles contributed to their prolonged release and enhanced absorption by cells. In addition, nanoparticles with curcumin showed increased cytotoxic activity in human glioblastoma tumor cell lines in vitro compared to free curcumin and were less toxic to healthy cells and embryos of *Danio rerio* fish. Thus, nanoparticles based on amphiphilic derivatives of poly-N-vinylpyrrolidone can be a promising delivery system for hydrophobic drugs. Zoya Saif et al. prepared risedronate-loaded mPEG-coated hydroxyapatite, thiolated chitosan-based nanoparticles (RIS-HA-TCS-mPEG) for the treatment of osteoporosis (Contribution 2). In vivo studies showed that, compared with non-coated RIS-HA-TCS, a marketed drug preparation and a plain risedronate pharmaceutical substance, RIS-HA-TCS-mPEG nanoparticles significantly inhibited bone loss by preventing bone resorption and promoted its regeneration. RIS-HA-TCS-mPEG also promoted the restoration of trabecular bone architecture and, when administered orally, this preparation demonstrated improved bioavailability and safety and was stable for more than 90 days. Thus, RIS-HA-TCS-mPEG shows pronounced potential in the treatment and prevention of osteoporosis, without significant side effects.

One of the key factors influencing the characteristics of nanocarriers is their method of synthesis, which determines the size, shape, stability, polydispersity, and sterility of the final product (Contribution 3). Mario Menéndez Miranda et al. synthesized polyvinyl alcohol-stabilized silver nanoparticles using radiolysis. The particles obtained using this method were homogeneous, sterile, and of high purity. The effect of various silver salt precursors on the properties and structure of the nanoparticles was also investigated. The nanoparticles effectively inhibited the growth of *Staphylococcus aureus* compared to silver nanoparticles obtained by other methods. Thus, radiolysis is a promising method for obtaining silver nanoparticles, which facilitates their widespread application in various industries.

Finally, the current Special Issue contains two reviews containing information on various nano-scaled drug delivery systems, their characteristics and properties, and their potential areas of application (Contributions 4, 5). Davoodbasha MubarakAli et al. reviewed the use of various stimuli-response nano-sized carriers for the treatment of diseases caused by bacteria (Contribution 4). The combined use of stimuli for effective bacteriostatic and bactericidal action was also shown. In the conclusion of the review, the authors talk about the difficulties of using stimuli-response nano-carriers in clinical practice and consider possible solutions. In the second review article, Maria V. Shestovskaya et al. consider the use of magnetic iron oxide nanoparticles as radiosensitizers for cancer therapy (Contribution 5). The review provides information on methods for modifying the surface of iron oxide nanoparticles to enhance their therapeutic effect, the mechanisms underlying the radiosensitizing effect, and the possibility of using them in combined chemoradiation therapy for cancer.

Thus, the research and review papers presented in this Special Issue highlight the diversity of the nanotechnology-based drug delivery systems developed to help solve complex problems in biomedicine. We thank all the authors of this Special Issue for their high-quality research and their contribution to such an interesting area of science and technology.

## Abbreviations

The following abbreviations are used in this manuscript:

DNA	Deoxyribonucleic acid
RNA	Ribonucleic acid
PEG	Polyethylene glycol
EPR	Enhanced permeability and retention
MPS	Mononuclear phagocytic system
